# Supplementary material for: Towards geospatially-resolved public-health surveillance via wastewater sequencing
Source: Nat Commun. 2024 Sep 27;15:8386. doi: 10.1038/s41467-024-52427-x (PMC11436780; doi:10.1038/s41467-024-52427-x)
Supplement: Supplementary file 1 — Supplementary Information [file 41467_2024_52427_MOESM1_ESM.pdf]

Supplementary Information

Towards geospatially-resolved public-health surveillance via wastewater sequencing

Supplementary Figure 1: A) Overlap between identified bacterial families in metatranscriptomic data by different classification approaches. B) The top 25 most abundant genera across the different taxonomic classification approaches. The relative abundance values are on the scale of 0 to 1 for Kraken2/Bracken and Xtree and 0 to 100 for MetaPhlAn4 (the default output of the software). Error bars represent standard deviations from mean. Source data are provided as a Source Data file.

Supplementary Figure 2: Results from the Microbiome Association Study illustrating bacterial expression and different wastewater-associated features. Volcano plots depicting the number of associated features and direction therein for different variables. X-axis is the beta-coefficient from the variable listed at the top of the plot. Y-axis is negative log(10) BY-adjusted p-value (i.e., q-value). Source data are provided as a Source Data file.

Supplementary Figure 3: The relative abundance of putative, low abundance pathogens bacteria as identified by A) Kraken2 and B) XTree. Heatmaps are hierarchically clustered. HOSP = hospital, DORM = dormitory, SCHOOL = primary/secondary school, WWTP = wastewater treatment plant, UC = university campus. Source data are provided as a Source Data file.

Supplementary Figure 4: Viral identification beta diversity, modeled after the bacterial beta diversity analysis in Figure 3. A) The top panel is the viral-species-specific beta diversity (bray curtis distance) between all samples within a given site. The line underneath corresponds to the average beta diversity at each associated site. The colored blocks on the bottom are vertically aligned with the population size points and the beta diversity dotplots, and colors correspond to the different sampling location types indicated in the legend. B) The intersections between different location types, with the bars indicating the intersection size and the black dots indicating the sites underneath being compared. These bars are vertically aligned with the middle and top panels, which show the relative abundance and prevalence, respectively, of all bacterial species represented by each bar. Source data are provided as a Source Data file.

Supplementary Figure 5: Results from the Microbiome Association Study illustrating viral family abundance and different wastewater-associated features. Volcano plots depicting the number of associated features and direction therein for different variables. X-axis is the beta-coefficient from the variable listed at the top of the plot. Y-axis is negative log(10) BY-adjusted p-value (i.e., q-value). Source data are provided as a Source Data file.

Supplementary Figure 6: The prevalence of different pathogen-associated genes in wastewater. Pathogen-associated genes are derived from the PathFinder database (see *Methods*). Source data are provided as a Source Data file.

Supplementary Figure 7: The top specific ARGs for different antibiotics across all sample types. Asterisks correspond to if a given ARG was enriched (in terms of log10 observations per 10k reads) in hospital wastewater when compared to all other sites according to the adjusted p-value on a t-test. P-values were adjusted by the Benjamini-Yekutieli procedure. For an asterisk to be present, an antibiotic class must have been enriched in all four comparisons (e.g., hospitals vs dormitories, hospitals vs the university campus, hospitals vs primary schools, and hospitals vs the wastewater treatment plant). Source data are provided as a Source Data file.

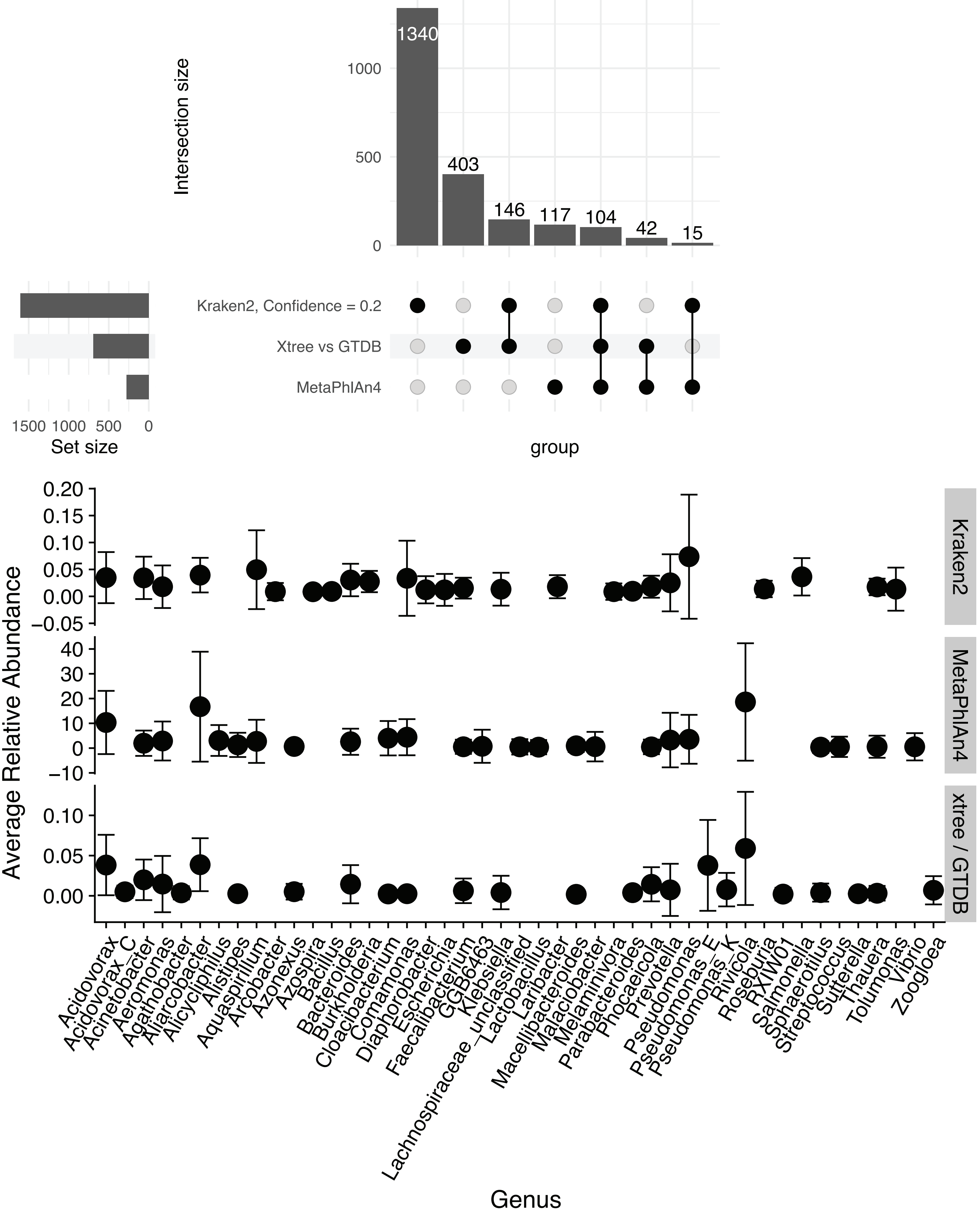

Intersection size
1340
403
146
117
104
42
15
Kraken2, Confidence = 0.2
Xtree vs GTDB
MetaPhlAn4
Set size
group
Average Relative Abundance
Kraken2
MetaPhlAn4
xtree / GTDB
Genus
Acidovorax
Acidovorax_C
Acinetobacter
Aeromonas
Agathobacter
Aliarcobacter
Alicycliphilus
Alistipes
Aquaspirillum
Arcobacter
Azonexus
Azospira
Bacillus
Bacteroides
Burkholderia
Cloacibacterium
Comamonas
Diaphorobacter
Escherichia
Faecalibacterium
GGB6463
Klebsiella
Lachnospiraceae_unclassified
Lactobacillus
Laribacter
Macellibacteroides
Malaciobacter
Melaminivora
Parabacteroides
Phocaeicola
Prevotella
Pseudomonas
Pseudomonas_E
Pseudomonas_K
Rivicola
Roseburia
RXIW01
Salmonella
Sphaerotilus
Streptococcus
Sutterella
Thauera
Tolumonas
Vibrio
Zoogloea

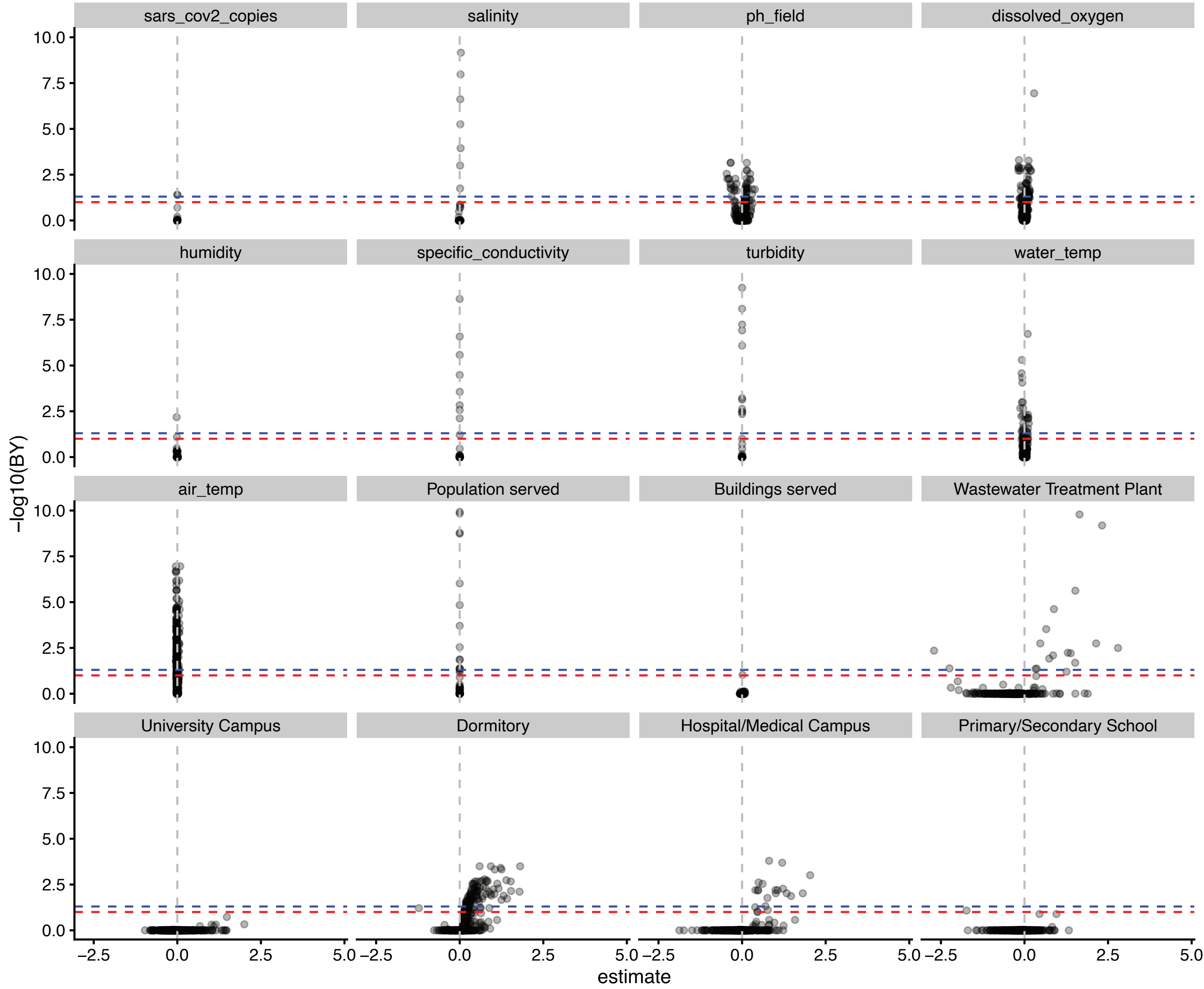

sars_cov2_copies
salinity
ph_field
dissolved_oxygen
humidity
specific_conductivity
turbidity
water_temp
air_temp
Population served
Buildings served
Wastewater Treatment Plant
University Campus
Dormitory
Hospital/Medical Campus
Primary/Secondary School
−log10(BY)
estimate
10.0
7.5
5.0
2.5
0.0
−2.5

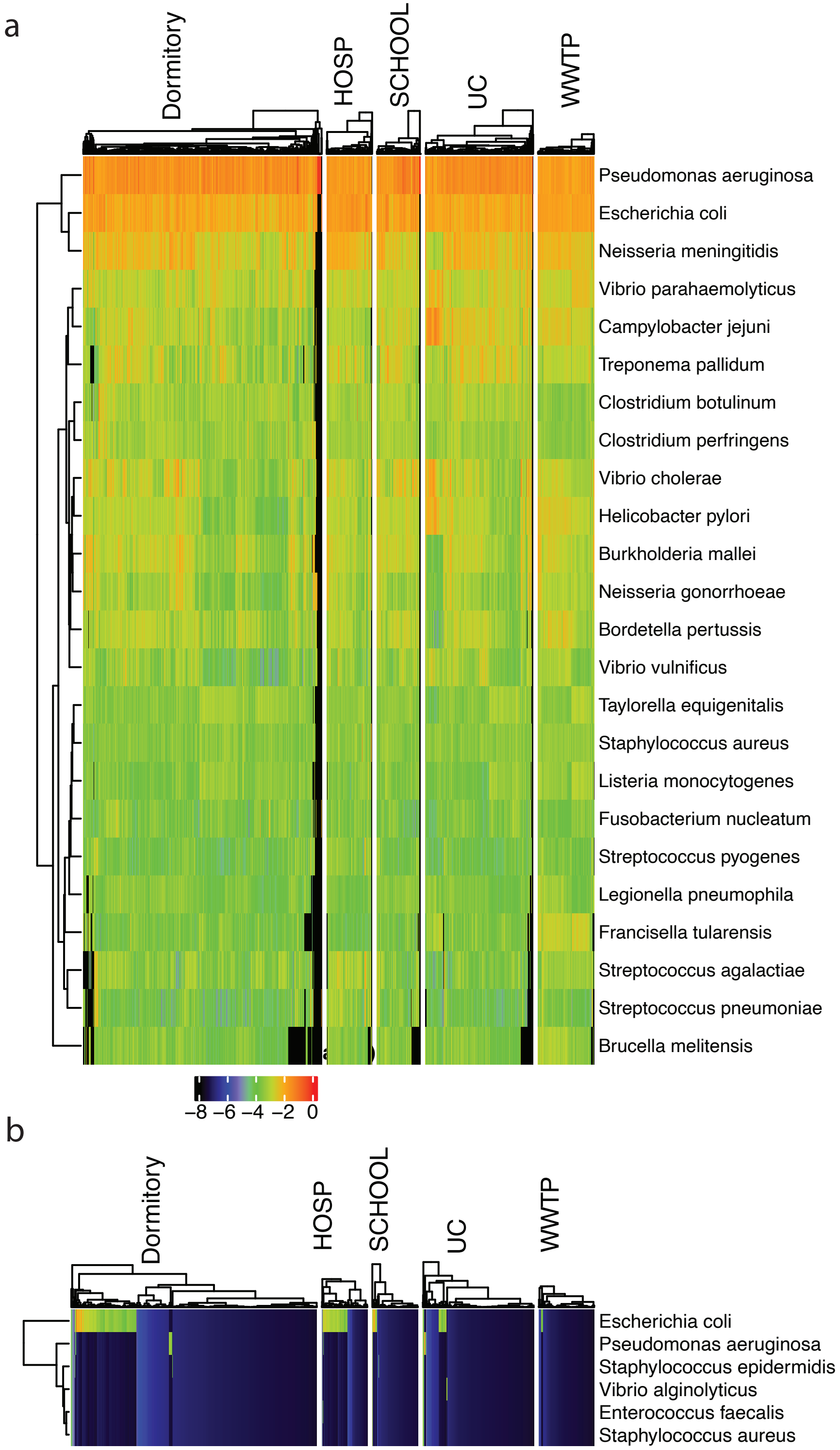
a
Dormitory
HOSP
SCHOOL
UC
WWTP
Pseudomonas aeruginosa
Escherichia coli
Neisseria meningitidis
Vibrio parahaemolyticus
Campylobacter jejuni
Treponema pallidum
Clostridium botulinum
Clostridium perfringens
Vibrio cholerae
Helicobacter pylori
Burkholderia mallei
Neisseria gonorrhoeae
Bordetella pertussis
Vibrio vulnificus
Taylorella equigenitalis
Staphylococcus aureus
Listeria monocytogenes
Fusobacterium nucleatum
Streptococcus pyogenes
Legionella pneumophila
Francisella tularensis
Streptococcus agalactiae
Streptococcus pneumoniae
Brucella melitensis
−8 −6 −4 −2 0
b
Dormitory
HOSP
SCHOOL
UC
WWTP
Escherichia coli
Pseudomonas aeruginosa
Staphylococcus epidermidis
Vibrio alginolyticus
Enterococcus faecalis
Staphylococcus aureus

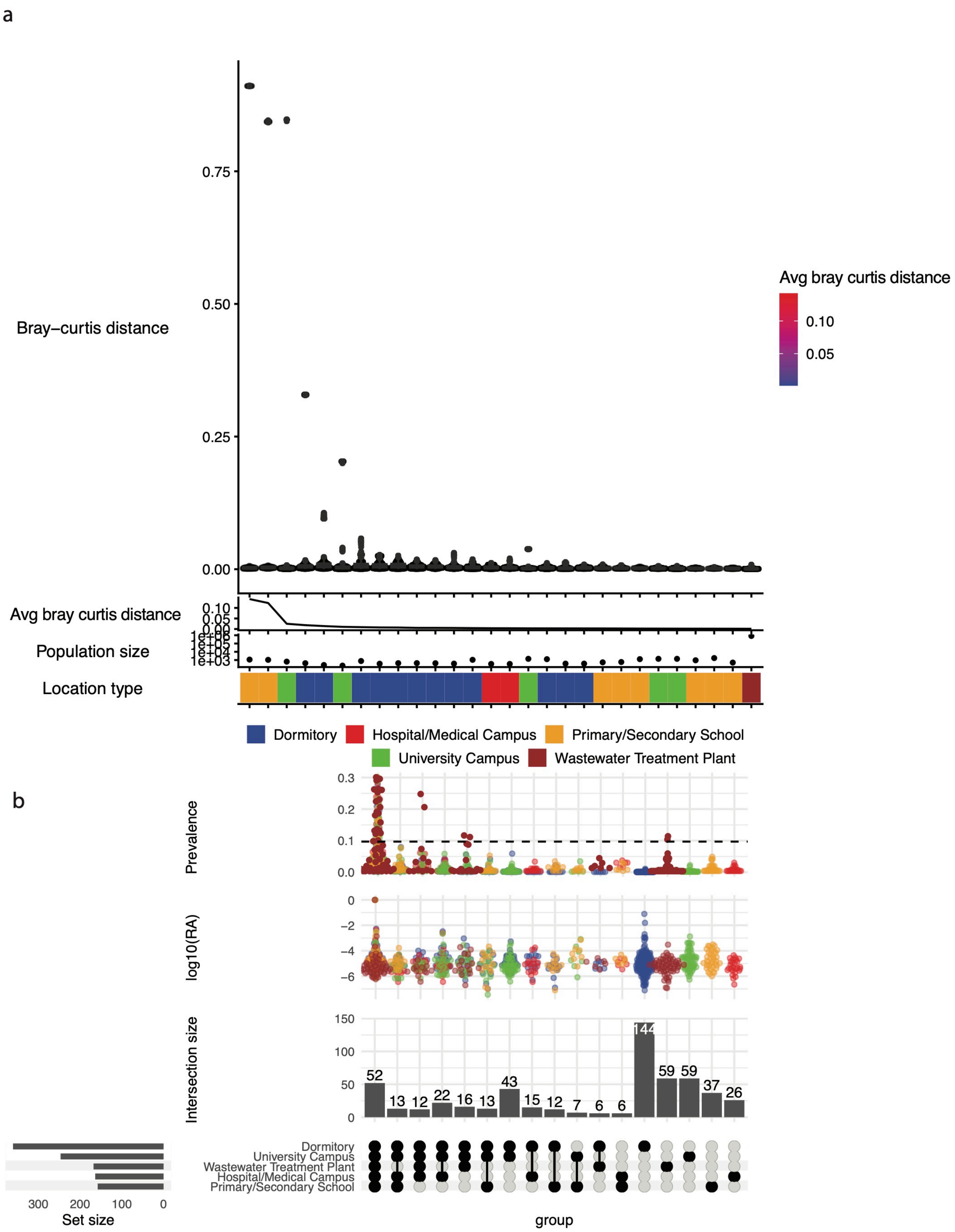
a
Bray–curtis distance
0.75
0.50
0.25
0.00
Avg bray curtis distance
0.10
0.05
Population size
Location type
Dormitory
Hospital/Medical Campus
Primary/Secondary School
University Campus
Wastewater Treatment Plant
b
Prevalence
log10(RA)
Intersection size
52
13
12
22
16
13
43
15
12
7
6
6
144
59
59
37
26
Dormitory
University Campus
Wastewater Treatment Plant
Hospital/Medical Campus
Primary/Secondary School
Set size
group

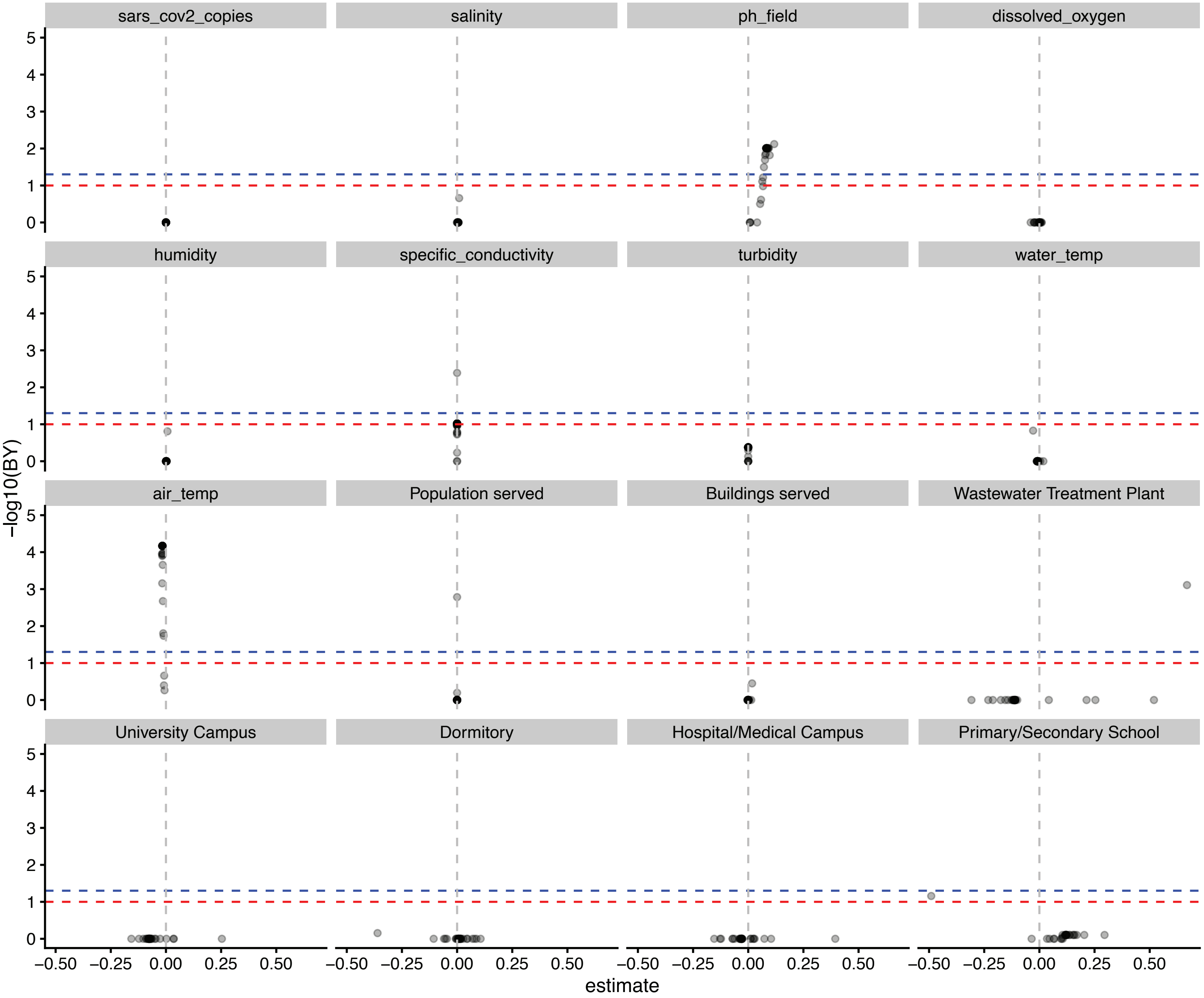

sars_cov2_copies
salinity
ph_field
dissolved_oxygen
humidity
specific_conductivity
turbidity
water_temp
air_temp
Population served
Buildings served
Wastewater Treatment Plant
University Campus
Dormitory
Hospital/Medical Campus
Primary/Secondary School
−log10(BY)
0
1
2
3
4
5
−0.50
−0.25
0.00
0.25
0.50
estimate

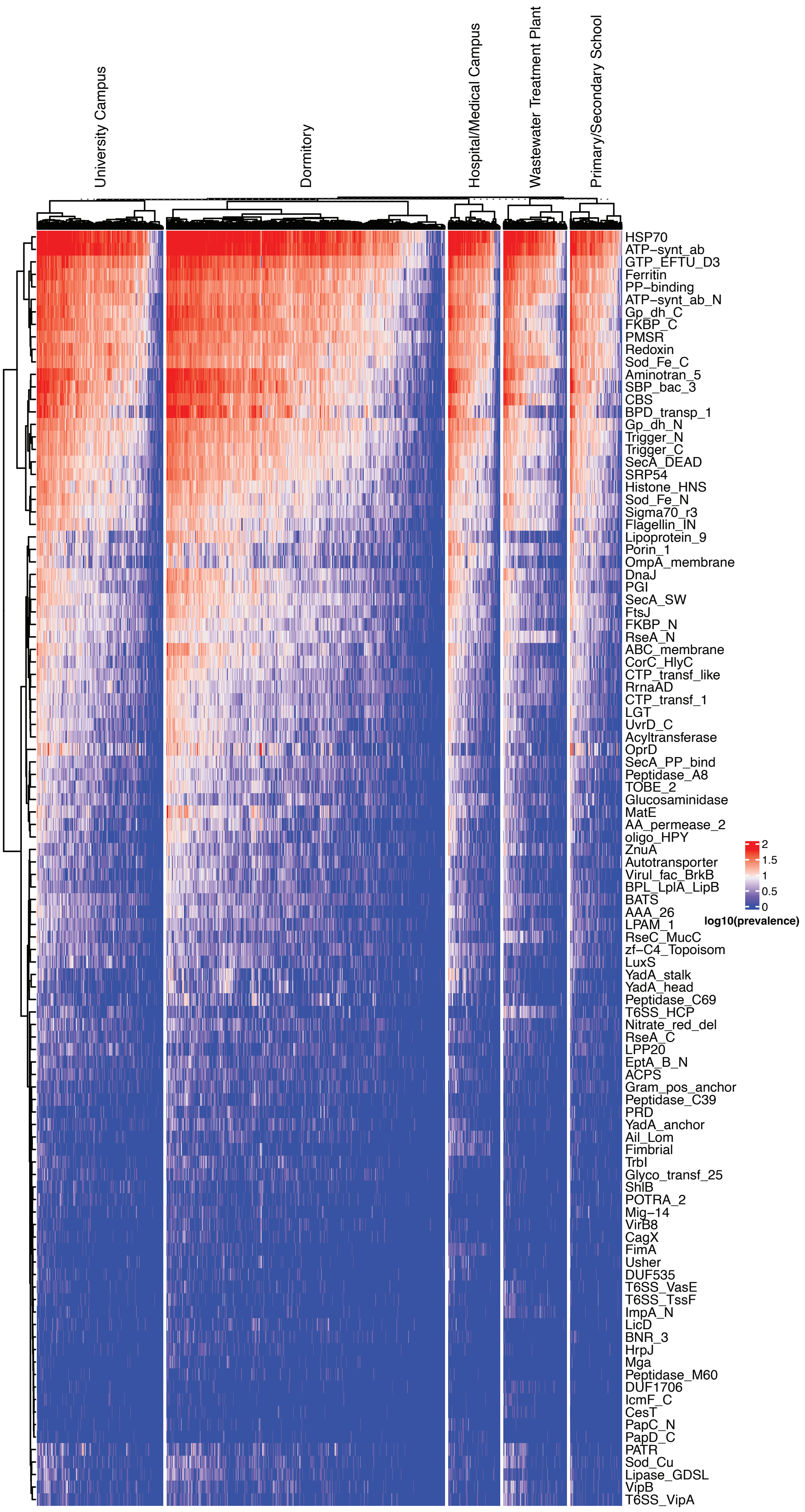
University Campus
Dormitory
Hospital/Medical Campus
Wastewater Treatment Plant
Primary/Secondary School
HSP70
ATP−synt_ab
GTP_EFTU_D3
Ferritin
PP−binding
ATP−synt_ab_N
Gp_dh_C
FKBP_C
PMSR
Redoxin
Sod_Fe_C
Aminotran_5
SBP_bac_3
CBS
BPD_transp_1
Gp_dh_N
Trigger_N
Trigger_C
SecA_DEAD
SRP54
Histone_HNS
Sod_Fe_N
Sigma70_r3
Flagellin_IN
Lipoprotein_9
Porin_1
OmpA_membrane
DnaJ
PGI
SecA_SW
FtsJ
FKBP_N
RseA_N
ABC_membrane
CorC_HlyC
CTP_transf_like
RrnaAD
CTP_transf_1
LGT
UvrD_C
Acyltransferase
OprD
SecA_PP_bind
Peptidase_A8
TOBE_2
Glucosaminidase
MatE
AA_permease_2
oligo_HPY
ZnuA
Autotransporter
Virul_fac_BrkB
BPL_LplA_LipB
BATS
AAA_26
LPAM_1
RseC_MucC
zf−C4_Topoisom
LuxS
YadA_stalk
YadA_head
Peptidase_C69
T6SS_HCP
Nitrate_red_del
RseA_C
LPP20
EptA_B_N
ACPS
Gram_pos_anchor
Peptidase_C39
PRD
YadA_anchor
Ail_Lom
Fimbrial
TrbI
Glyco_transf_25
ShlB
POTRA_2
Mig−14
VirB8
CagX
FimA
Usher
DUF535
T6SS_VasE
T6SS_TssF
ImpA_N
LicD
BNR_3
HrpJ
Mga
Peptidase_M60
DUF1706
IcmF_C
CesT
PapC_N
PapD_C
PATR
Sod_Cu
Lipase_GDSL
VipB
T6SS_VipA
2
1.5
1
0.5
0
log10(prevalence)

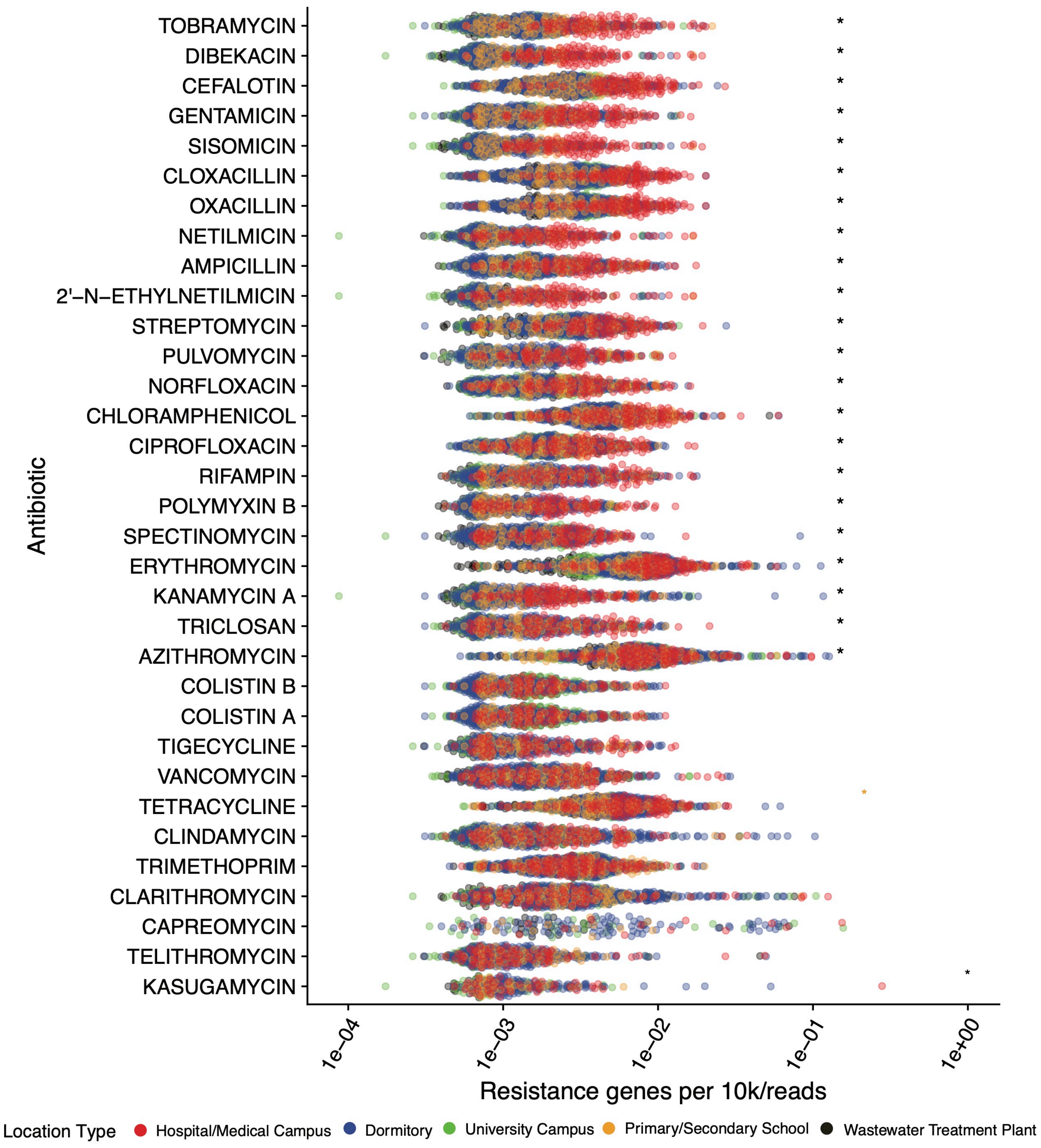

Antibiotic
TOBRAMYCIN
DIBEKACIN
CEFALOTIN
GENTAMICIN
SISOMICIN
CLOXACILLIN
OXACILLIN
NETILMICIN
AMPICILLIN
2'−N−ETHYLNETILMICIN
STREPTOMYCIN
PULVOMYCIN
NORFLOXACIN
CHLORAMPHENICOL
CIPROFLOXACIN
RIFAMPIN
POLYMYXIN B
SPECTINOMYCIN
ERYTHROMYCIN
KANAMYCIN A
TRICLOSAN
AZITHROMYCIN
COLISTIN B
COLISTIN A
TIGECYCLINE
VANCOMYCIN
TETRACYCLINE
CLINDAMYCIN
TRIMETHOPRIM
CLARITHROMYCIN
CAPREOMYCIN
TELITHROMYCIN
KASUGAMYCIN
1e−04
1e−03
1e−02
1e−01
1e+00
Resistance genes per 10k/reads
Location Type
Hospital/Medical Campus
Dormitory
University Campus
Primary/Secondary School
Wastewater Treatment Plant
