# Supplementary material for: Towards geospatially-resolved public-health surveillance via wastewater sequencing
Source: Nat Commun. 2024 Sep 27;15:8386. doi: 10.1038/s41467-024-52427-x (PMC11436780; doi:10.1038/s41467-024-52427-x)
Supplement: Supplementary file 2 — Reporting Summary [file 41467_2024_52427_MOESM2_ESM.pdf]

nature portfolio

Corresponding author(s): Christopher E Mason

Last updated by author(s): Aug 30, 2024

Reporting Summary

Nature Portfolio wishes to improve the reproducibility of the work that we publish. This form provides structure for consistency and transparency in reporting. For further information on Nature Portfolio policies, see our Editorial Policies and the Editorial Policy Checklist.

Statistics

For all statistical analyses, confirm that the following items are present in the figure legend, table legend, main text, or Methods section.

n/a	Confirmed	
☐	☒	The exact sample size (*n*) for each experimental group/condition, given as a discrete number and unit of measurement
☐	☒	A statement on whether measurements were taken from distinct samples or whether the same sample was measured repeatedly
☐	☒	The statistical test(s) used AND whether they are one- or two-sided<br>*Only common tests should be described solely by name; describe more complex techniques in the Methods section.*
☐	☒	A description of all covariates tested
☐	☒	A description of any assumptions or corrections, such as tests of normality and adjustment for multiple comparisons
☐	☒	A full description of the statistical parameters including central tendency (e.g. means) or other basic estimates (e.g. regression coefficient) AND variation (e.g. standard deviation) or associated estimates of uncertainty (e.g. confidence intervals)
☐	☒	For null hypothesis testing, the test statistic (e.g. *F*, *t*, *r*) with confidence intervals, effect sizes, degrees of freedom and *P* value noted<br>*Give P values as exact values whenever suitable.*
☒	☐	For Bayesian analysis, information on the choice of priors and Markov chain Monte Carlo settings
☒	☐	For hierarchical and complex designs, identification of the appropriate level for tests and full reporting of outcomes
☐	☒	Estimates of effect sizes (e.g. Cohen's *d*, Pearson's *r*), indicating how they were calculated

Our web collection on statistics for biologists contains articles on many of the points above.

Software and code

Policy information about availability of computer code

Data collection	No custom software was used in data collection.
Data analysis	Kraken2 V2.1.2, seqtk V1.3-r106, bwa V0.7.17-r1188, sambamba 0.7.0, ivar V1.13, bedtools genomecov V2.30.0, mosdepth V0.3.3, lofreq V2.1.5, VEP V104.3, Freyja V1.4.2, bbtools suite V38.92, MetaPhlan4 V4.0.4, Xtree V0.92i, decontam R package 1.14.0, MetaViralSPAdes V3.15.5, CheckV V0.7.0, Prodigal-gv V2.6.3, hmmsearch V3.4, mmseqs2 V15.6f452, RGI V6.0.3, BLAST 2.15.0+, MAFFT V7.520, TrimAl V1.2, RAxML-ng v1.2.1, R V4.1.3, ggtree V3.7.2, ggplot2 V3.4.2, tidyverse V2.0.0, ComplexHeatmap V2.10.0, UpSetR V1.4.0, ComplexUpSet V1.3.3, phytools V1.5-1, ape V5.7-1, umap V0.2.10.0, ggbeeswarm V0.7.1, tidytext V0.4.1, circlize V0.4.15, vegan V2.6-4, broom V1.0.4, reshape2 V1.4.4, ggpubr V0.6.0<br><br>Code is available at https://github.com/b-tierney/radx-wastewater-scripts.

For manuscripts utilizing custom algorithms or software that are central to the research but not yet described in published literature, software must be made available to editors and reviewers. We strongly encourage code deposition in a community repository (e.g. GitHub). See the Nature Portfolio guidelines for submitting code & software for further information.

Data

Policy information about availability of data

All manuscripts must include a data availability statement. This statement should provide the following information, where applicable:

- Accession codes, unique identifiers, or web links for publicly available datasets
- A description of any restrictions on data availability
- For clinical datasets or third party data, please ensure that the statement adheres to our policy

In compliance with the NIH RADx-rad Data Coordination Center (DCC) requirements, the raw sequencing data was submitted to the Sequence Read Archive (SRA). The wastewater samples were annotated with the rich metadata and the sequencing information (fastq files) was included in the submission. The submitted data can be found in the SRA under the accession PRJNA946141. Source data are provided with this paper. Patient data used in Figure 1 are described in the following manuscript, Carattini et al(Carattini et al. 2023). Furthermore, the metadata associated with the wastewater samples' sequencing data was extracted from the Illumina operational files, validated, organized, and submitted to the NIH data hub via DCC [https://radx-hub.nih.gov/home], where the SF-RAD data is associated with the dbGaP study accession phs002525.v1.p1. The metadata standards specifications used to describe the data were developed in collaboration with the SF-RAD members and the DCC and formally defined and registered at FAIRsharing.org. Additional processed files (e.g., taxonomic abundance matrices) are available at https://figshare.com/projects/Geospatially-resolved_public-health_surveillance_via_wastewater_sequencing/198412.

Research involving human participants, their data, or biological material

Policy information about studies with human participants or human data. See also policy information about sex, gender (identity/presentation), and sexual orientation and race, ethnicity and racism.

Reporting on sex and gender	N/A
Reporting on race, ethnicity, or other socially relevant groupings	N/A
Population characteristics	N/A
Recruitment	N/A
Ethics oversight	The sharing of clinical data for internal research purposes was approved by the University of Miami IRB (IRB ID: 20210164, MOD000010222). In addition, at the county level, the Florida Department of Health documented positive COVID-19 detections by zip code and this data was provided to the research team through delegation of the above IRB. Given the availability of clinically-based data, the wastewater sample collection program was designed to provide a population match between clinical data and populations contributing wastewater to a specific wastewater sampling location. The clinical data described in this study was collected and published as part of another study (https://www.mdpi.com/1999-4915/15/3/593). The data itself was collected without an IRB (as the samples were permanently de-identified and unlinked from subjects and used for surveillance purposes only), however our team decided to include extant data itself in our proposed wastewater IRB. It was approved by UMiami IRB (IRB Study Number: 20210464, Version 10). We did not get informed consent for clinical data because the data was provided after the fact and published elsewhere. The approving IRB determined informed consent was not necessary for these samples.

Note that full information on the approval of the study protocol must also be provided in the manuscript.

Field-specific reporting

Please select the one below that is the best fit for your research. If you are not sure, read the appropriate sections before making your selection.

☐ Life sciences ☐ Behavioural & social sciences ☒ Ecological, evolutionary & environmental sciences

For a reference copy of the document with all sections, see nature.com/documents/nr-reporting-summary-flat.pdf

Ecological, evolutionary & environmental sciences study design

All studies must disclose on these points even when the disclosure is negative.

Study description	Wastewater samples were collected and sequenced over two years in Miami Dade County.
Research sample	Research samples were wastewater collected directly from sewer lines. Each samples represents the collective sewage waste from the population upstream of said sewer lines.
Sampling strategy	No sample size calculation was performed; samples were collected based on ability to access sites.
Data collection	Sample collection volumes were 2 liters with the first liter being used for water quality analyses in the field (pH, water temperature, dissolved oxygen, specific conductivity, and turbidity). Sampling data was recorded electronically by manuscript authors.
Timing and spatial scale	Samples were collected weekly from September 30, 2020 through September 21, 2022. Sampling sites included sewer holes from

	residential dormitories (14 sites), sites representing major portions of campus (4 sites, 3 corresponding to the main residential campus and 1 corresponding to the university marine campus with no residences), from laboratory/administrative buildings at the medical campus (2 sites), from the University hospital (2 sites), grade school sites (8 locations representing 9 grade schools, 3 high schools, 2 middle schools, and 4 elementary schools with one elementary school and one middle school discharging to the same sewer hole), and from a regional wastewater treatment plant (Central District) serving a population of 830,000 from Miami-Dade County. In addition, at three sites both grab and composite samples were collected accounting for the balance of the 34 sampling sites. Between September 2021 and January 2022, samples were collected two-times per week at the dormitory sites to provide additional data for on-campus mitigation measures.<br><br>Composite samples were collected at the building and cluster scale using either an ISCO 6712 autosampler (time-paced) or at the community scale wastewater treatment plant using an HACH autosampler (flow-paced). All other samples were collected using a grab sampling technique, due to limited availability of multiple autosamplers.
Data exclusions	No data were excluded.
Reproducibility	Outside of collecting multiple samples from the same sites over two years, no explicit attempts at reproducibility were made.
Randomization	This study was not a randomized trial.
Blinding	This study was not blinded.

Did the study involve field work? ☐ Yes ☒ No

Reporting for specific materials, systems and methods

We require information from authors about some types of materials, experimental systems and methods used in many studies. Here, indicate whether each material, system or method listed is relevant to your study. If you are not sure if a list item applies to your research, read the appropriate section before selecting a response.

Materials & experimental systems

n/a	Involved in the study
☒	☐ Antibodies
☒	☐ Eukaryotic cell lines
☒	☐ Palaeontology and archaeology
☒	☐ Animals and other organisms
☒	☐ Clinical data
☒	☐ Dual use research of concern
☒	☐ Plants

Methods

n/a	Involved in the study
☒	☐ ChIP-seq
☒	☐ Flow cytometry
☒	☐ MRI-based neuroimaging

Plants

Seed stocks	*Report on the source of all seed stocks or other plant material used. If applicable, state the seed stock centre and catalogue number. If plant specimens were collected from the field, describe the collection location, date and sampling procedures.*
Novel plant genotypes	*Describe the methods by which all novel plant genotypes were produced. This includes those generated by transgenic approaches, gene editing, chemical/radiation-based mutagenesis and hybridization. For transgenic lines, describe the transformation method, the number of independent lines analyzed and the generation upon which experiments were performed. For gene-edited lines, describe the editor used, the endogenous sequence targeted for editing, the targeting guide RNA sequence (if applicable) and how the editor was applied.*
Authentication	*Describe any authentication procedures for each seed stock used or novel genotype generated. Describe any experiments used to assess the effect of a mutation and, where applicable, how potential secondary effects (e.g. second site T-DNA insertions, mosiacism, off-target gene editing) were examined.*
