# Supplementary material for: Towards geospatially-resolved public-health surveillance via wastewater sequencing
Source: Nat Commun. 2024 Sep 27;15:8386. doi: 10.1038/s41467-024-52427-x (PMC11436780; doi:10.1038/s41467-024-52427-x)
Supplement: Supplementary file 3 — Description of Additional Supplementary Files [file 41467_2024_52427_MOESM3_ESM.pdf]

Supplementary Data 1: Results from SARS-CoV-2 VoC identification. In the final tab, the three p-values for the Spearman correlations were generated from two-sided tests and not adjusted for multiple testing.

Supplementary Data 2: Taxa removed during the sample decontamination process (see *Methods*).

Supplementary Data 3: Bulk sequencing summary statistics, including read counts, summarized CheckV output, and assembly statistics.

Supplementary Data 4: Raw association output between microbial taxonomic features and wastewater characteristics. Two-sided p-values were generated via Satterthwaite's approximation method. We report nominal and adjusted (via both Benjamini-Hochberg [BH] and Benjamini-Yekutieli [BY]) p-values.
